# The low-temperature phase of morpholinium tetra­fluoro­borate

**DOI:** 10.1107/S1600536808004339

**Published:** 2008-03-05

**Authors:** Magdalena Owczarek, Przemyslaw Szklarz, Ryszard Jakubas, Tadeusz Lis

**Affiliations:** a Faculty of Chemistry, University of Wrocław, F. Joliot-Curie 14, 50-383 Wrocław, Poland

## Abstract

The crystal structure of the low-temperature form of the title compound, C_4_H_10_NO^+^·BF_4_^−^, was determined at 80 K. Two reversible phase transitions, at 158/158 and 124/126 K (heating/cooling), were detected by differential scanning calorimetry for this compound, and the sequence of phase transitions was subsequently confirmed by single-crystal X-ray diffraction experiments. The asymmetric unit at 80 K consists of three BF_4_^−^ tetra­hedral anions and three morpholinium cations (*Z*′ = 3). Hydrogen-bonded morpholinium cations form chains along the [100] direction. The BF_4_^−^ anions are connected to these chains by N—H⋯F hydrogen bonds. In the crystal structure, two different layers perpendicular to the [001] direction can be distinguished, which differ in the geometry of the hydrogen bonds between cationic and anionic species.

## Related literature

For the crystal structures of morpholinium chlorate(VII) (isostructural with the title compound) and morpholinium hydrogensulfate, see: Grigoriev *et al.* (2008 ▶); Yin *et al.* (2006 ▶).
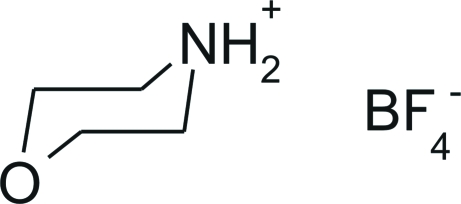


## Experimental

### 

#### Crystal data

C_4_H_10_NO^+^·BF_4_^−^
*M* *_r_* = 174.94
Orthorhombic, 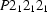

*a* = 8.106 (4) Å
*b* = 9.417 (4) Å
*c* = 28.572 (11) Å
*V* = 2181.0 (16) Å^3^
*Z* = 12
Mo *K*α radiation
μ = 0.17 mm^−1^
*T* = 80 (2) K
0.5 × 0.5 × 0.4 mm

#### Data collection

Kuma KM-4 CCD κ-geometry diffractometer
Absorption correction: none
20616 measured reflections
4642 independent reflections
3913 reflections with *I* > 2σ(*I*)
*R*_int_ = 0.033

#### Refinement

*R*[*F* ^2^ > 2σ(*F* ^2^)] = 0.037
*wR*(*F* ^2^) = 0.092
*S* = 1.13
4642 reflections
298 parameters
H-atom parameters constrained
Δρ_max_ = 0.46 e Å^−3^
Δρ_min_ = −0.31 e Å^−3^

### 

Data collection: *CrysAlis CCD* (Oxford Diffraction, 2006 ▶); cell refinement: *CrysAlis CCD*; data reduction: *CrysAlis RED* (Oxford Diffraction, 2006 ▶); program(s) used to solve structure: *SHELXS97* (Sheldrick, 2008 ▶); program(s) used to refine structure: *SHELXL97* (Sheldrick, 2008 ▶); molecular graphics: *DIAMOND* (Brandenburg, 1998 ▶); software used to prepare material for publication: *SHELXL97*.

## Supplementary Material

Crystal structure: contains datablocks I, global. DOI: 10.1107/S1600536808004339/gk2132sup1.cif

Structure factors: contains datablocks I. DOI: 10.1107/S1600536808004339/gk2132Isup2.hkl

Additional supplementary materials:  crystallographic information; 3D view; checkCIF report

## Figures and Tables

**Table 1:** Hydrogen-bond geometry (Å, °)

*D*—H⋯*A*	*D*—H	H⋯*A*	*D*⋯*A*	*D*—H⋯*A*
N1—H1*C*⋯F2	0.92	1.96	2.742 (2)	142
N1—H1*D*⋯O3^i^	0.92	1.96	2.857 (2)	164
N2—H2*C*⋯F8	0.92	1.96	2.799 (2)	151
N2—H2*D*⋯O2^ii^	0.92	1.95	2.842 (2)	164
N3—H3*C*⋯F9	0.92	1.96	2.742 (2)	141
N3—H3*D*⋯O1^i^	0.92	1.96	2.856 (2)	164

Symmetry codes: (i) 
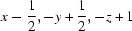
; (ii) 
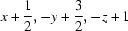
.

**Fig. 1.**
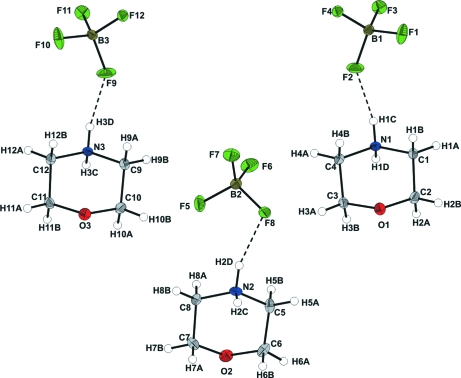
Asymmetric unit of the title compound with atom labelling scheme. The displacement ellipsoids were drawn at the 50% probability level.

**Fig. 2.**
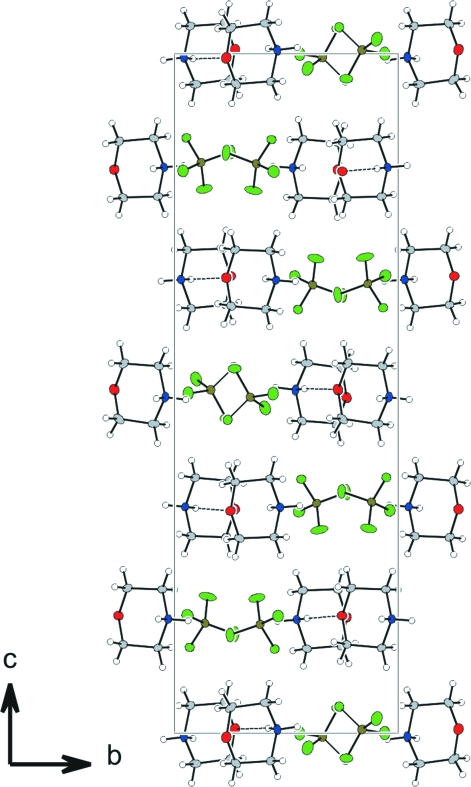
Projection of the crystal packing along [100].

**Fig. 3.**
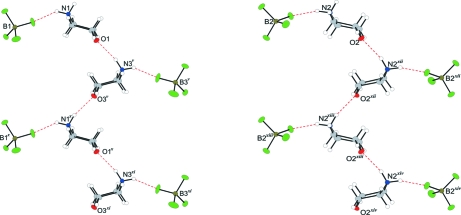
Two types of chains in the crystal structure of (I). Symmetry codes: (v) 1/2 + x, 1/2 - y, 1 - z; (x) 1 + x, y, z; (xi) 3/2 + x, 1/2 - y,1 - z; (xii) -1/2 + x, 3/2 - y,1 - z; (xiii) -1 + x, y, z; (xiv) -3/2 + x, 3/2 - y, 1 - z.

## supplementary crystallographic information

### Comment

The morpholinium tetrafluoroborate (I) undergoes two reversible phase transitions at 158/158 K and 124/126 K (heating/cooling). At the room temperature it crystallizes in the orthorhombic space group *Pnma* with *Z*'=1. The intermediate phase appeared to be incommensurately modulated. The structure of (I) in the low-temperature phase contains ordered BF_4_^-^ tetrahedral units and morpholinium cations in the chair conformation. The bond distances and angles in the BF_4_^-^ anions and morpholinium cations are in agreement with the expected values. The N–H morpholinium protons are involved in the hydrogen bonds N–H···O (morpholine-morpholine zigzag chains) and N—H···F with BF_4_^-^ anions. The tetrahedral BF_4_^-^ anions occupy voids between morpholinium chains.

The title compound I appeared to be isostructural with morpholinium chlorate(VII) at 100 K (Grigoriev *et al., *2008). Both structures are characterized by two independent hydrogen bonded layers and only slight differences in geometry of hydrogen bonds between morpholinium and anionic species are observed.

The room-temperature phase of I is isostructural with the morpholinium hydrogensulfate (Yin *et al., *2006). The tetrafluoroborate anions appear to be dynamically disordered in this phase. During the phase transition from modulated to the low temperature phase at 124 K threefold increase of the lattice parameter b is observed.

### Experimental

The title compound was prepared by reaction of stoichiometric amounts of morpholine and concentrated tetrafluoroboric acid in water. The resulting solid was recrystallized from methanol at room temperature. The crystal for X-ray measurements was slowly cooled from room temperature to 80 K. During cooling, the crystal undergoes phase transition from centrosymmetric (*Pnma*), through modulated phase, to the non-centrosymmetric *P*2_1_2_1_2_1_ space group.

### Refinement

In the absence of signifiant anomalous scattering effects, Friedel pairs were averaged. All H atoms were found in difference-Fourier maps. In the final refinement, all H atoms were positioned geometrically and treated as riding on their parent atoms, with C–H distances of 0.99 Å and N–H distances of 0.92 Å, and with *U*_iso_(H) values of 1.2*U*_eq_(C) and 1.2*U*_eq_(N).

### Crystal data

**Table:**

C_4_H_10_NO^+^·BF_4_^–^	*F*_000_ = 1080
*M**_r_* = 174.94	*D*_x_ = 1.598 Mg m^−^^3^
Orthorhombic, *P*2_1_2_1_2_1_	Mo *K*α radiation λ = 0.71073 Å
Hall symbol: P 2ac 2ab	Cell parameters from 14871 reflections
*a* = 8.106 (4) Å	θ = 5–34º
*b* = 9.417 (4) Å	µ = 0.17 mm^−^^1^
*c* = 28.572 (11) Å	*T* = 80 (2) K
*V* = 2181.0 (16) Å^3^	Block, colorless
*Z* = 12	0.5 × 0.5 × 0.4 mm

### Data collection

**Table:**

Kuma KM-4 CCD κ-geometry diffractometer	3913 reflections with *I* > 2σ(*I*)
Radiation source: fine-focus sealed tube	*R*_int_ = 0.033
Monochromator: graphite	θ_max_ = 34.3º
*T* = 80(2) K	θ_min_ = 4.8º
ω scans	*h* = −12→9
Absorption correction: none	*k* = −14→14
20616 measured reflections	*l* = −44→32
4642 independent reflections	

### Refinement

**Table:**

Refinement on *F*^2^	Secondary atom site location: difference Fourier map
Least-squares matrix: full	Hydrogen site location: inferred from neighbouring sites
*R*[*F*^2^ > 2σ(*F*^2^)] = 0.037	H-atom parameters constrained
*wR*(*F*^2^) = 0.092	*w* = 1/[σ^2^(*F*_o_^2^) + (0.052*P*)^2^] where *P* = (*F*_o_^2^ + 2*F*_c_^2^)/3
*S* = 1.13	(Δ/σ)_max_ = 0.001
4642 reflections	Δρ_max_ = 0.46 e Å^−^^3^
298 parameters	Δρ_min_ = −0.31 e Å^−^^3^
Primary atom site location: structure-invariant direct methods	Extinction correction: none

### Special details

**Table:**

Geometry. All e.s.d.'s (except the e.s.d. in the dihedral angle between two l.s. planes) are estimated using the full covariance matrix. The cell e.s.d.'s are taken into account individually in the estimation of e.s.d.'s in distances, angles and torsion angles; correlations between e.s.d.'s in cell parameters are only used when they are defined by crystal symmetry. An approximate (isotropic) treatment of cell e.s.d.'s is used for estimating e.s.d.'s involving l.s. planes.
Refinement. Refinement of *F*^2^ against ALL reflections. The weighted *R*-factor *wR* and goodness of fit *S* are based on *F*^2^, conventional *R*-factors *R* are based on *F*, with *F* set to zero for negative *F*^2^. The threshold expression of *F*^2^ > σ(*F*^2^) is used only for calculating *R*-factors(gt) *etc*. and is not relevant to the choice of reflections for refinement. *R*-factors based on *F*^2^ are statistically about twice as large as those based on *F*, and *R*- factors based on ALL data will be even larger.

### Fractional atomic coordinates and isotropic or equivalent isotropic displacement parameters (Å^2^)

**Table:**

	*x*	*y*	*z*	*U*_iso_*/*U*_eq_	
B1	0.5748 (2)	−0.38148 (19)	0.66006 (6)	0.0138 (3)	
B2	0.38324 (17)	0.16152 (18)	0.50547 (6)	0.0145 (3)	
B3	0.5721 (2)	−0.36435 (19)	0.33817 (6)	0.0137 (3)	
F1	0.67871 (11)	−0.36736 (13)	0.69879 (3)	0.0272 (2)	
F2	0.52308 (13)	−0.24786 (11)	0.64441 (4)	0.0285 (2)	
F3	0.43715 (11)	−0.46227 (11)	0.67231 (3)	0.0207 (2)	
F4	0.66156 (10)	−0.44725 (10)	0.62384 (3)	0.01512 (17)	
F5	0.35554 (13)	0.23160 (12)	0.46328 (3)	0.0258 (2)	
F6	0.24001 (11)	0.09601 (11)	0.52149 (4)	0.0257 (2)	
F7	0.50716 (11)	0.06012 (10)	0.50017 (4)	0.0244 (2)	
F8	0.43192 (12)	0.26312 (10)	0.53898 (3)	0.01994 (19)	
F9	0.52566 (14)	−0.23018 (12)	0.35451 (4)	0.0333 (3)	
F10	0.67387 (11)	−0.35204 (14)	0.29913 (3)	0.0306 (3)	
F11	0.43177 (11)	−0.44221 (11)	0.32671 (3)	0.0222 (2)	
F12	0.65993 (10)	−0.43246 (10)	0.37371 (3)	0.01573 (18)	
O1	0.69421 (11)	0.25053 (12)	0.67290 (4)	0.0142 (2)	
O2	0.23260 (11)	0.76931 (12)	0.49397 (4)	0.0166 (2)	
O3	0.69339 (11)	0.26920 (12)	0.32955 (4)	0.0151 (2)	
N1	0.45612 (14)	0.02870 (13)	0.66772 (4)	0.0111 (2)	
H1C	0.4314	−0.0664	0.6654	0.013*	
H1D	0.3585	0.0784	0.6690	0.013*	
N2	0.45949 (13)	0.54141 (13)	0.50542 (4)	0.0130 (2)	
H2C	0.4778	0.4455	0.5086	0.016*	
H2D	0.5599	0.5868	0.5063	0.016*	
N3	0.45585 (14)	0.04709 (13)	0.33294 (4)	0.0113 (2)	
H3C	0.4306	−0.0480	0.3350	0.014*	
H3D	0.3586	0.0971	0.3311	0.014*	
C1	0.55316 (17)	0.05500 (16)	0.71154 (5)	0.0133 (3)	
H1A	0.6538	−0.0046	0.7117	0.016*	
H1B	0.4861	0.0300	0.7393	0.016*	
C2	0.60018 (17)	0.21069 (18)	0.71332 (5)	0.0154 (3)	
H2A	0.4989	0.2693	0.7149	0.018*	
H2B	0.6659	0.2291	0.7419	0.018*	
C3	0.59955 (16)	0.22934 (17)	0.63068 (5)	0.0139 (3)	
H3A	0.6654	0.2594	0.6032	0.017*	
H3B	0.4985	0.2883	0.6318	0.017*	
C4	0.55267 (18)	0.07415 (16)	0.62565 (5)	0.0137 (3)	
H4A	0.4855	0.0607	0.5970	0.016*	
H4B	0.6535	0.0155	0.6228	0.016*	
C5	0.35391 (17)	0.59281 (18)	0.54488 (5)	0.0160 (3)	
H5A	0.4122	0.5791	0.5750	0.019*	
H5B	0.2498	0.5379	0.5459	0.019*	
C6	0.31647 (17)	0.74878 (17)	0.53769 (5)	0.0179 (3)	
H6A	0.2465	0.7838	0.5636	0.022*	
H6B	0.4206	0.8038	0.5378	0.022*	
C7	0.33543 (18)	0.72648 (17)	0.45575 (5)	0.0166 (3)	
H7A	0.4384	0.7830	0.4560	0.020*	
H7B	0.2777	0.7450	0.4258	0.020*	
C8	0.37715 (17)	0.56993 (17)	0.45929 (5)	0.0149 (3)	
H8A	0.2751	0.5126	0.4567	0.018*	
H8B	0.4517	0.5426	0.4334	0.018*	
C9	0.54945 (18)	0.09205 (16)	0.37573 (5)	0.0140 (3)	
H9A	0.6502	0.0335	0.3791	0.017*	
H9B	0.4803	0.0782	0.4039	0.017*	
C10	0.59621 (16)	0.24721 (16)	0.37110 (5)	0.0147 (3)	
H10A	0.4950	0.3059	0.3695	0.018*	
H10B	0.6601	0.2771	0.3989	0.018*	
C11	0.60280 (17)	0.22966 (17)	0.28841 (5)	0.0153 (3)	
H11A	0.6707	0.2484	0.2603	0.018*	
H11B	0.5014	0.2878	0.2862	0.018*	
C12	0.55689 (17)	0.07375 (16)	0.29006 (5)	0.0132 (3)	
H12A	0.4929	0.0480	0.2618	0.016*	
H12B	0.6580	0.0148	0.2908	0.016*	

### Atomic displacement parameters (Å^2^)

**Table:**

	*U*^11^	*U*^22^	*U*^33^	*U*^12^	*U*^13^	*U*^23^
B1	0.0138 (6)	0.0131 (8)	0.0143 (7)	−0.0009 (6)	−0.0009 (5)	−0.0013 (6)
B2	0.0134 (5)	0.0142 (7)	0.0158 (7)	0.0010 (5)	−0.0013 (5)	−0.0011 (7)
B3	0.0145 (6)	0.0128 (8)	0.0139 (7)	−0.0011 (6)	−0.0002 (5)	0.0014 (6)
F1	0.0194 (4)	0.0478 (7)	0.0145 (4)	−0.0065 (5)	−0.0016 (3)	−0.0079 (5)
F2	0.0373 (5)	0.0128 (5)	0.0353 (6)	0.0082 (4)	0.0082 (4)	0.0009 (4)
F3	0.0151 (4)	0.0218 (5)	0.0251 (4)	−0.0049 (4)	0.0030 (4)	0.0001 (4)
F4	0.0160 (4)	0.0152 (4)	0.0142 (4)	0.0024 (3)	0.0004 (3)	−0.0005 (4)
F5	0.0338 (5)	0.0305 (6)	0.0131 (4)	0.0110 (5)	−0.0033 (4)	0.0005 (4)
F6	0.0173 (4)	0.0230 (5)	0.0369 (5)	−0.0078 (4)	0.0042 (4)	−0.0056 (5)
F7	0.0200 (4)	0.0197 (5)	0.0334 (5)	0.0079 (3)	0.0017 (4)	0.0011 (5)
F8	0.0288 (4)	0.0143 (5)	0.0167 (4)	−0.0052 (4)	−0.0058 (3)	0.0007 (4)
F9	0.0418 (6)	0.0122 (5)	0.0458 (6)	0.0099 (5)	−0.0112 (5)	−0.0032 (5)
F10	0.0210 (5)	0.0571 (8)	0.0137 (4)	−0.0115 (5)	0.0012 (3)	0.0090 (5)
F11	0.0148 (4)	0.0249 (5)	0.0270 (5)	−0.0056 (4)	−0.0027 (4)	0.0019 (5)
F12	0.0179 (4)	0.0160 (4)	0.0133 (4)	0.0026 (3)	−0.0011 (3)	0.0001 (4)
O1	0.0122 (4)	0.0166 (5)	0.0138 (4)	−0.0041 (4)	−0.0018 (3)	0.0007 (4)
O2	0.0128 (3)	0.0165 (5)	0.0205 (5)	0.0050 (4)	−0.0019 (4)	−0.0021 (5)
O3	0.0126 (4)	0.0160 (5)	0.0166 (5)	−0.0042 (4)	0.0008 (3)	−0.0005 (4)
N1	0.0105 (5)	0.0092 (5)	0.0136 (5)	−0.0006 (4)	−0.0003 (4)	−0.0004 (5)
N2	0.0112 (4)	0.0094 (5)	0.0185 (6)	0.0002 (4)	−0.0001 (4)	0.0012 (5)
N3	0.0113 (5)	0.0096 (5)	0.0130 (5)	−0.0005 (4)	0.0011 (4)	0.0011 (5)
C1	0.0156 (6)	0.0139 (7)	0.0105 (5)	−0.0015 (5)	−0.0007 (5)	−0.0001 (6)
C2	0.0165 (6)	0.0172 (7)	0.0124 (6)	−0.0027 (5)	−0.0005 (4)	−0.0031 (6)
C3	0.0152 (6)	0.0147 (7)	0.0116 (6)	−0.0018 (5)	−0.0009 (4)	0.0012 (6)
C4	0.0176 (6)	0.0129 (7)	0.0105 (5)	−0.0011 (5)	0.0009 (5)	−0.0016 (6)
C5	0.0139 (5)	0.0237 (8)	0.0104 (5)	−0.0020 (6)	−0.0014 (5)	−0.0003 (6)
C6	0.0158 (6)	0.0199 (8)	0.0182 (6)	0.0020 (6)	−0.0018 (5)	−0.0080 (6)
C7	0.0170 (6)	0.0169 (7)	0.0159 (6)	0.0017 (5)	0.0001 (5)	0.0053 (6)
C8	0.0175 (6)	0.0152 (7)	0.0120 (6)	0.0014 (5)	0.0019 (5)	−0.0019 (6)
C9	0.0167 (6)	0.0144 (7)	0.0109 (5)	0.0009 (5)	−0.0001 (5)	0.0012 (6)
C10	0.0157 (6)	0.0141 (7)	0.0144 (6)	−0.0009 (5)	0.0004 (4)	−0.0019 (6)
C11	0.0161 (6)	0.0162 (7)	0.0135 (6)	−0.0027 (5)	0.0014 (4)	0.0019 (6)
C12	0.0140 (5)	0.0153 (7)	0.0103 (5)	−0.0011 (5)	0.0008 (5)	−0.0010 (6)

### Geometric parameters (Å, °)

**Table:**

B1—F3	1.3950 (19)	C1—C2	1.516 (2)
B1—F4	1.3964 (19)	C1—H1A	0.9900
B1—F1	1.3971 (18)	C1—H1B	0.9900
B1—F2	1.400 (2)	C2—H2A	0.9900
B2—F6	1.3921 (18)	C2—H2B	0.9900
B2—F5	1.3926 (19)	C3—C4	1.517 (2)
B2—F7	1.3942 (18)	C3—H3A	0.9900
B2—F8	1.4098 (19)	C3—H3B	0.9900
B3—F10	1.3919 (18)	C4—H4A	0.9900
B3—F11	1.3926 (19)	C4—H4B	0.9900
B3—F12	1.3962 (19)	C5—C6	1.514 (2)
B3—F9	1.399 (2)	C5—H5A	0.9900
O1—C2	1.4338 (18)	C5—H5B	0.9900
O1—C3	1.4435 (17)	C6—H6A	0.9900
O2—C7	1.4318 (18)	C6—H6B	0.9900
O2—C6	1.4354 (18)	C7—C8	1.516 (2)
O3—C11	1.4350 (18)	C7—H7A	0.9900
O3—C10	1.4397 (18)	C7—H7B	0.9900
N1—C4	1.4968 (18)	C8—H8A	0.9900
N1—C1	1.4992 (17)	C8—H8B	0.9900
N1—H1C	0.9200	C9—C10	1.515 (2)
N1—H1D	0.9200	C9—H9A	0.9900
N2—C5	1.4960 (18)	C9—H9B	0.9900
N2—C8	1.5014 (18)	C10—H10A	0.9900
N2—H2C	0.9200	C10—H10B	0.9900
N2—H2D	0.9200	C11—C12	1.515 (2)
N3—C12	1.4950 (17)	C11—H11A	0.9900
N3—C9	1.4998 (18)	C11—H11B	0.9900
N3—H3C	0.9200	C12—H12A	0.9900
N3—H3D	0.9200	C12—H12B	0.9900
			
F3—B1—F4	110.30 (13)	H3A—C3—H3B	108.1
F3—B1—F1	109.61 (13)	N1—C4—C3	109.29 (11)
F4—B1—F1	109.00 (12)	N1—C4—H4A	109.8
F3—B1—F2	109.32 (13)	C3—C4—H4A	109.8
F4—B1—F2	108.22 (13)	N1—C4—H4B	109.8
F1—B1—F2	110.37 (14)	C3—C4—H4B	109.8
F6—B2—F5	111.11 (12)	H4A—C4—H4B	108.3
F6—B2—F7	109.46 (13)	N2—C5—C6	109.05 (12)
F5—B2—F7	110.28 (12)	N2—C5—H5A	109.9
F6—B2—F8	108.12 (12)	C6—C5—H5A	109.9
F5—B2—F8	108.14 (13)	N2—C5—H5B	109.9
F7—B2—F8	109.69 (11)	C6—C5—H5B	109.9
F10—B3—F11	109.85 (13)	H5A—C5—H5B	108.3
F10—B3—F12	108.61 (13)	O2—C6—C5	110.10 (12)
F11—B3—F12	110.21 (13)	O2—C6—H6A	109.6
F10—B3—F9	110.60 (14)	C5—C6—H6A	109.6
F11—B3—F9	109.52 (13)	O2—C6—H6B	109.6
F12—B3—F9	108.03 (13)	C5—C6—H6B	109.6
C2—O1—C3	110.75 (10)	H6A—C6—H6B	108.2
C7—O2—C6	110.50 (10)	O2—C7—C8	110.66 (12)
C11—O3—C10	110.98 (10)	O2—C7—H7A	109.5
C4—N1—C1	110.43 (11)	C8—C7—H7A	109.5
C4—N1—H1C	109.6	O2—C7—H7B	109.5
C1—N1—H1C	109.6	C8—C7—H7B	109.5
C4—N1—H1D	109.6	H7A—C7—H7B	108.1
C1—N1—H1D	109.6	N2—C8—C7	109.38 (12)
H1C—N1—H1D	108.1	N2—C8—H8A	109.8
C5—N2—C8	110.45 (10)	C7—C8—H8A	109.8
C5—N2—H2C	109.6	N2—C8—H8B	109.8
C8—N2—H2C	109.6	C7—C8—H8B	109.8
C5—N2—H2D	109.6	H8A—C8—H8B	108.2
C8—N2—H2D	109.6	N3—C9—C10	109.12 (12)
H2C—N2—H2D	108.1	N3—C9—H9A	109.9
C12—N3—C9	110.09 (11)	C10—C9—H9A	109.9
C12—N3—H3C	109.6	N3—C9—H9B	109.9
C9—N3—H3C	109.6	C10—C9—H9B	109.9
C12—N3—H3D	109.6	H9A—C9—H9B	108.3
C9—N3—H3D	109.6	O3—C10—C9	110.34 (12)
H3C—N3—H3D	108.2	O3—C10—H10A	109.6
N1—C1—C2	108.65 (11)	C9—C10—H10A	109.6
N1—C1—H1A	110.0	O3—C10—H10B	109.6
C2—C1—H1A	110.0	C9—C10—H10B	109.6
N1—C1—H1B	110.0	H10A—C10—H10B	108.1
C2—C1—H1B	110.0	O3—C11—C12	110.59 (12)
H1A—C1—H1B	108.3	O3—C11—H11A	109.5
O1—C2—C1	111.08 (12)	C12—C11—H11A	109.5
O1—C2—H2A	109.4	O3—C11—H11B	109.5
C1—C2—H2A	109.4	C12—C11—H11B	109.5
O1—C2—H2B	109.4	H11A—C11—H11B	108.1
C1—C2—H2B	109.4	N3—C12—C11	108.82 (11)
H2A—C2—H2B	108.0	N3—C12—H12A	109.9
O1—C3—C4	110.22 (12)	C11—C12—H12A	109.9
O1—C3—H3A	109.6	N3—C12—H12B	109.9
C4—C3—H3A	109.6	C11—C12—H12B	109.9
O1—C3—H3B	109.6	H12A—C12—H12B	108.3
C4—C3—H3B	109.6		
			
C4—N1—C1—C2	56.30 (14)	C6—O2—C7—C8	61.49 (15)
C3—O1—C2—C1	61.00 (14)	C5—N2—C8—C7	54.94 (14)
N1—C1—C2—O1	−58.22 (14)	O2—C7—C8—N2	−57.26 (14)
C2—O1—C3—C4	−60.55 (14)	C12—N3—C9—C10	57.02 (14)
C1—N1—C4—C3	−56.75 (15)	C11—O3—C10—C9	60.49 (15)
O1—C3—C4—N1	58.12 (14)	N3—C9—C10—O3	−58.07 (14)
C8—N2—C5—C6	−55.96 (14)	C10—O3—C11—C12	−60.89 (14)
C7—O2—C6—C5	−62.45 (15)	C9—N3—C12—C11	−57.07 (14)
N2—C5—C6—O2	59.27 (14)	O3—C11—C12—N3	58.66 (14)

### Hydrogen-bond geometry (Å, °)

**Table:**

*D*—H···*A*	*D*—H	H···*A*	*D*···*A*	*D*—H···*A*
N1—H1C···F2	0.92	1.96	2.742 (2)	142
N1—H1D···O3^i^	0.92	1.96	2.857 (2)	164
N2—H2C···F8	0.92	1.96	2.799 (2)	151
N2—H2D···O2^ii^	0.92	1.95	2.842 (2)	164
N3—H3C···F9	0.92	1.96	2.742 (2)	141
N3—H3D···O1^i^	0.92	1.96	2.856 (2)	164
C1—H1B···F1^iii^	0.99	2.42	3.261 (2)	143
C2—H2B···F10^iv^	0.99	2.39	3.337 (2)	160
C3—H3A···F5^v^	0.99	2.45	3.413 (2)	165
C5—H5A···F4^vi^	0.99	2.47	3.384 (2)	154
C5—H5B···F7^i^	0.99	2.54	3.410 (2)	147
C6—H6A···F12^i^	0.99	2.38	3.318 (2)	158
C8—H8B···F12^vi^	0.99	2.41	3.352 (2)	159
C9—H9B···F5	0.99	2.45	3.233 (2)	136
C11—H11A···F1^vii^	0.99	2.41	3.373 (2)	163
C12—H12A···F10^viii^	0.99	2.40	3.237 (2)	142
C12—H12B···F3^ix^	0.99	2.54	3.429 (2)	149

Symmetry codes: (i) *x*−1/2, −*y*+1/2, −*z*+1; (ii) *x*+1/2, −*y*+3/2, −*z*+1; (iii) −*x*+1, *y*+1/2, −*z*+3/2; (iv) −*x*+3/2, −*y*, *z*+1/2; (v) *x*+1/2, −*y*+1/2, −*z*+1; (vi) *x*, *y*+1, *z*; (vii) −*x*+3/2, −*y*, *z*−1/2; (viii) −*x*+1, *y*+1/2, −*z*+1/2; (ix) *x*+1/2, −*y*−1/2, −*z*+1.
